# Excessive activation of JAK-STAT signaling contributes to inflammation induced by acute *Vibrio* infection in shrimp

**DOI:** 10.1080/21505594.2025.2451169

**Published:** 2025-01-17

**Authors:** Hongliang Zuo, Xiya Yang, Youxi Wang, Bangping Hu, Zhiming Zhu, Zhixun Guo, Shaoping Weng, Jianguo He, Xiaopeng Xu

**Affiliations:** a State Key Laboratory of Biocontrol, School of Life Sciences/Southern Marine Science and Engineering Guangdong Laboratory (Zhuhai)/China-ASEAN Belt and Road Joint Laboratory on Mariculture Technology, Sun Yat-sen University, Guangzhou, P. R. China; b Institute of Aquatic Economic Animals and Guangdong Province Key Laboratory for Aquatic Economic Animals, Sun Yat-sen University, Guangzhou, P. R. China; c Provincial Observation and Research Station for Marine Ranching in Lingdingyang bay, Zhuhai, P. R. China; d South China Sea Fisheries Research Institute (CAFS), Guangzhou, P. R. China

**Keywords:** Shrimp, *Vibrio parahaemolyticus*, acute infection, inflammation, JAK-STAT pathway, immune response

## Abstract

Uncontrolled immune responses resulting from overactivated cellular signaling pathways, leading to inflammation and tissue injury, are a major cause of death in pathogen-infected individuals. This phenomenon has been well studied in mammals but is less explored in invertebrates. Bacteria of the genus *Vibrio* are among the most harmful pathogens to humans and aquatic animals. In shrimp, *Vibrio* infection is generally characterized by the sudden onset of disease, with pathological signs of opaque and whitish muscle tissue. The current study shows that shrimp acutely infected with high dose of *Vibrio parahaemolyticus* develop inflammation-like pathological changes, leading to rapid death. Excessive activation of JAK-STAT signaling, rather than the Dorsal and Relish pathways, results in overactivation of shrimp immunity and is a major cause of inflammation induced by acute *Vibrio* infection. Weakening JAK-STAT signaling attenuates the inflammatory response and reduces mortality caused by acute *Vibrio* infection in shrimp, whereas enhancing JAK-STAT signaling can convert a normal infection into an acute one, accelerating shrimp death. Therefore, this study indicates that, similar to that in mammals, the pathogenesis of infectious diseases in invertebrates is complicated by inflammatory responses triggered by dysregulated immune signaling.

## Introduction

The pathogenesis of infectious diseases is generally complicated by pathogen virulence and host immune response intensity. Inadequate immune response promotes infection, while excessive activation of immunity leads to tissue injury and death [1]. For instance, in bacterial infection, overactivation of immune cells and overproduction of cytokines critically result in inflammation and sepsis [2,3]. In the COVID-19 disease, the cytokine storm induced is known to be associated with death [4–6]. Overactivation of the immune system is intrinsically triggered by excessive activation of multiple cellular signaling pathways, among which the JAK-STAT and NF-κB pathways are the most concerned [7–10]. The role of immune overactivation and its underlying signaling mechanisms in the pathogenesis of infectious diseases has been extensively studied in mammals but remains poorly understood in invertebrates.

The *Vibrio* genus of Gram-negative bacteria, ubiquitously distributed in estuarine and marine environments worldwide, is a representative group of aquatic pathogens most harmful to human and aquatic animals [11,12]. Vibriosis caused by *Vibrio* infection occurs widely in crustaceans, bringing huge economic losses to aquaculture industry [13,14]. In most cases, *Vibrio* concentrations in water reach extremely high levels, leading to acute infection characterized by sudden or rapid onset of diseases in shrimp, in which opacity and whitening of muscle tissue are the most common pathological features [15,16]. However, the mechanisms underlying the pathogenic and lethal effects of *Vibrio* on invertebrates in the context of acute infection remain unclear.

Pacific white shrimp, *Penaeus vannamei*, is currently the most cultured shrimp worldwide. In recent years, the global shrimp farming industry is heavily threatened by bacterial infectious diseases [17]. *Vibrio parahaemolyticus*, a representative species of the genus *Vibrio*, is one of the major pathogens of *P. vannamei*. *V. parahaemolyticus* carrying the virulence genes *pirA*^*vp*^ and *pirB*^*vp*^ is capable of causing acute hepatopancreatic necrosis disease (AHPND), leading to widespread mortality in farmed shrimp [18,19]. This has resulted in significant economic losses in the global shrimp aquaculture industry in recent years.

Like other invertebrates, shrimp possess the JAK-STAT pathway with a regulatory mechanism similar to that of vertebrates, in which activation of the transcription factor STAT is dependent on JAK-mediated tyrosine phosphorylation [20–23]. The *P. vannamei* JAK-STAT pathway consists of the cellular receptor Domeless, the kinase JAK, the transcription factor STAT, the inhibitors SOCS2 and PIAS, and the activator PTPN6 [24–28]. Activation of JAK-STAT signaling is important for mounting the immune defense against low- or medium-dose bacterial infection in shrimp [29–31], but its role in acute infection remains unconcerned.

In this study, we demonstrated that immune overactivation is also a major cause of shrimp death from *V. parahaemolyticus* acute infection. Excessive activation of STAT, but not the NF-κB family members Dorsal and Relish, is the major cause of immune activation in *V. parahaemolyticus*-infected shrimp. These findings are important for understanding the pathogenesis of vibriosis in crustaceans and helpful to reveal the occurring mechanisms of acute bacterial infection in invertebrates. Based on these, we suggest that appropriate attenuation, rather than enhancement, of shrimp immunity could be applicable to the treatment of acute bacterial infections.

## Materials and methods

### Animal and pathogen

Healthy *P. vannamei* (~10 g) from a shrimp farm in Zhuhai, China, was acclimated for at least 1 week at ~27 °C a 400-liter triple-aquarium circulation system. The water was continuously sterilized using a UV sterilization filtration system to eliminate pathogenic microorganisms. The system containing air-pumped seawater with a salinity of 5 ppt, and the shrimps were fed an artificial diet at 3% of body weight, divided into two meals per day. The *V. parahaemolyticus* strain with *pirA*^*vp*^ and *pirB*^*vp*^ genes was obtained from shrimp with AHPND in an aquaculture farm in Zhanjiang, China. The *V. parahaemolyticus* stock was serially diluted and spread on thiosulfate-citrate-bile salts-sucrose (TCBS) agar plates, followed by overnight incubation at 37°C. Individual bacterial colonies on the plates were counted, and the colony-forming units (CFU) of the stock solution were calculated based on the dilution factor. *V. parahaemolyticus* was cultured to logarithmic phase as stocks at 3 × 10^9^ CFU/mL phosphate buffer solution (PBS, pH 7.4) as previously described [32].

### RNA interference and quantitative real-time RT-PCR

Based on RNA interference (RNAi) strategy, expression of *JAK*, *STAT*, and *SOCS2* was suppressed by injection with specific dsRNA synthesized using a T7 RiboMAX™ Express RNAi System (Promega, USA). Briefly, templates for each gene were amplified using specific primers containing T7 polymerase promoter tags (Table S1). Following the manufacturer’s protocol, two single-strand RNAs were synthesized and annealed to a double-stranded RNA. The dsRNA specific to green fluorescent protein (GFP) gene was synthesized as control. Shrimps were injected at the second abdominal segment with 10 μg dsRNA diluted in 50 μL PBS or PBS (as control). At 48 h post injection, hemolymph was extracted from nine shrimps using a 2.0 mL syringe containing 1 mL anticoagulant buffer (25 mm citric acid, 51 mm trisodium citrate, and 80 mm glucose) and immediately centrifuged at 1,000 × g for 4 min at 4 °C to collect hemocytes. Gills were simultaneously sampled as well. To assess the efficiency of RNAi, the expression of *JAK*, *STAT*, and *SOCS2* in hemocytes and gills was analyzed using quantitative real-time RT-PCR (qRT-PCR). In brief, total RNA was purified and reverse-transcribed into cDNA using a PrimeScript RT reagent kit with gDNA Eraser (Takara, Japan) according to the manufacturer’s protocols. qRT-PCR was performed in a 10-μL mixture containing 1 μL cDNA, 5 μL 2 × SYBR premix ExTaq II (Takara, Japan), and 500 nM each primer (Table S1) on a LightCycle 480 System (Roche, Switzerland). The optimized thermal cycling parameters were 95°C for 1 min, 40 cycles of 95°C for 10 s and 60°C for 30 s. Melting curves were generated by increasing the temperature from 65°C to 95°C at 0.5°C/s. Gene expression levels were calculated using the 2^−ΔΔCt^ method and normalized to that of the internal control elongation factor 1 alpha gene (*EF-1α*, GenBank accession number: GU136229). Experiments were repeated using three different batches of shrimp, and representative results are shown.

### Immune challenge

Immune challenge experiments were performed in triplicate, and the collected samples were used for subsequent qRT-PCR, Western-blot, flow cytometry, and immunofluorescence analyses. For the acute *V. parahaemolyticus* infection analysis, shrimp were divided into five groups (*n* = 30) and intramuscularly injected with 50 μL PBS (control), 3 × 10^6^, 1.5 × 10^6^, 5 × 10^5^, or 3 × 10^5^ CFU of *V. parahaemolyticus*. The mortality was recorded every 2 h. In a parallel experiment, gill tissues from six surviving shrimp in each group were randomly sampled at 8 and 16 h post-bacterial challenge to quantify bacterial content. To investigate the role of the JAK-STAT signaling pathway in acute infection, shrimp (*n* = 40) were injected with gene-specific dsRNA specific to *JAK*, *STAT*, *SOCS2*, or the control *GFP* genes, as described previously, and were infected 48 h later with 1.5 × 10^6^ or 5 × 10^5^ CFU *V. parahaemolyticus*. Mortality was recorded for each group. In a parallel experiment, gill tissues from six surviving shrimp per group were randomly sampled at 8 and 16-h post-bacterial challenge to quantify bacterial content. Muscle tissue from injection sites was paraffin-sectioned and stained with hematoxylin-eosin (HE) for pathological analysis. Total DNA extraction was performed using the DNeasy Blood & Tissue Kit (Qiagen, Germany). The bacterial load in gill tissues was assessed by relative qPCR targeting the *pirA*^*vp*^ toxin gene (GenBank accession no. KP324996), with the *P. vannamei EF-1α* gene (GenBank accession no. GU136229) serving as the internal control.

### Western-blot analysis

Total nuclear proteins of hemocytes (each sample pooled from 30 shrimp) were extracted using NE-PER Nuclear and Cytoplasmic Extraction Reagents (Thermo, USA) for Western-blot analysis using rabbit anti-shrimp STAT, Dorsal and Relish antibodies (GL Biochem, China). The antibodies against histone H3 (CST, USA) and β-actin (MBL, Japan) were used to detect the nuclear and cytoplasmic internal control proteins, respectively. For dimmer protein analysis, hemocytes were treated with immunoprecipitation lysis buffer (Thermo, USA), separated using native-PAGE, and translocated into NC membrane for Western-blot analysis. The gray values of the specific protein bands were calculated using Quantity one 4.6.2 software (Bio-Rad, USA) by Gauss model and normalized to those of internal control proteins.

### Flow cytometry

The reactive oxygen species (ROS) levels, apoptosis rates, and phagocytic activities of hemocytes in *V. parahaemolyticus*-infected shrimp or *STAT-*, *JAK-* and *SOCS2*-silenced shrimp were examined by flow cytometry. Each sample was collected from nine shrimps and washed with 2 × Leibovitz’s L-15 medium (Gibco, USA) triply.

For ROS analysis, cells were added with 2,7-dichlorodi-hydrofluorescein diacetate (DCFH-DA) to a final concentration of 10 μmol/L and incubated at 37 °C for 20 min. After washing, cells were detected using C6 flow cytometer (BD, USA).

The apoptosis of hemocytes was detected using Annexin V-FITC apoptosis detection kit (Sigma, USA). In brief, after washing twice with Dulbecco’s PBS, cells were resuspended with 500 μL 1 × binding buffer at ~1 × 10^6^ cells/mL, added with 5 μL of Annexin V FITC conjugate and 10 μL of propidium iodide (PI), incubated at room temperature for 10 min in dark, and detected using flow cytometry.

For phagocytosis analysis, fluorescein isothiocyanate (FITC)-labled *V. parahaemolyticus* was prepared. Briefly, *V. parahaemolyticus* was cultured to the logarithmic phase and harvested by centrifugation at 5,000 × g at room temperature for 10 min. After washing twice with PBS, the bacteria were resuspended in carbonate buffer solution (0.5 mol/L, pH 9.5) to a concentration of 1 × 10^9^ CFU/mL, followed by the addition of FITC to a final concentration of 50 μg/mL. The mixture was incubated at room temperature with gentle mixing for 2 h. The bacteria were then harvested by centrifugation at 5,000 × g at 4°C for 10 min and washed twice with PBS. The FITC-labeled bacteria were fixed with 0.1% paraformaldehyde for 30 min at 37°C, washed twice with PBS, and stored at −20°C away from light. Hemocytes were stained with Dil (Beyotime, China), mixed with FITC-labled *V. parahaemolyticus* at 1:100 ratio, and incubated at 28 °C for 1 h. After washing three times with L-15 medium, hemocytes were detected using flow cytometry for the Dil and FITC signals. The thresholds and boundaries of cells that have phagocytized bacteria were set based on the detection of the controls Dil-stained hemocytes and FITC-labeled *V. parahaemolyticus* phagocytized by unstained cells (Supplementary Fig S1). A total of 500,000 events were detected for each sample.

### Immunofluorescence

After fixation with 4% paraformaldehyde for 10 min and treatment with 1% Triton X-100 for 20 min, hemolymph smears on siliconized slides were successively incubated with rabbit antibodies against shrimp STAT, Dorsal and Relish (GL Biochem, China), mouse antibody against β-actin (MBL, Japan), Alexa fluor 488 conjugated goat anti-rabbit antibody (Abcam, USA), and Alexa fluor 594 conjugate goat anti-mouse antibody (CST, USA). After staining with Hoechst 33342 (Invitrogen, USA) for the nuclei, slides were observed using a Leica LSM 410 confocal microscope (Germany).

### Statistics

Statistical comparisons were performed by two-tailed unpaired Student’s t test or one-way ANOVA followed by Dunnett’s post hoc test. The survival data were analyzed by Kaplan–Meier log-rank χ^2^ tests. *p* < 0.05 was considered to be statistically significant.

## Results

### Acute V. parahaemolyticus infection in shrimp

**Figure 1.**
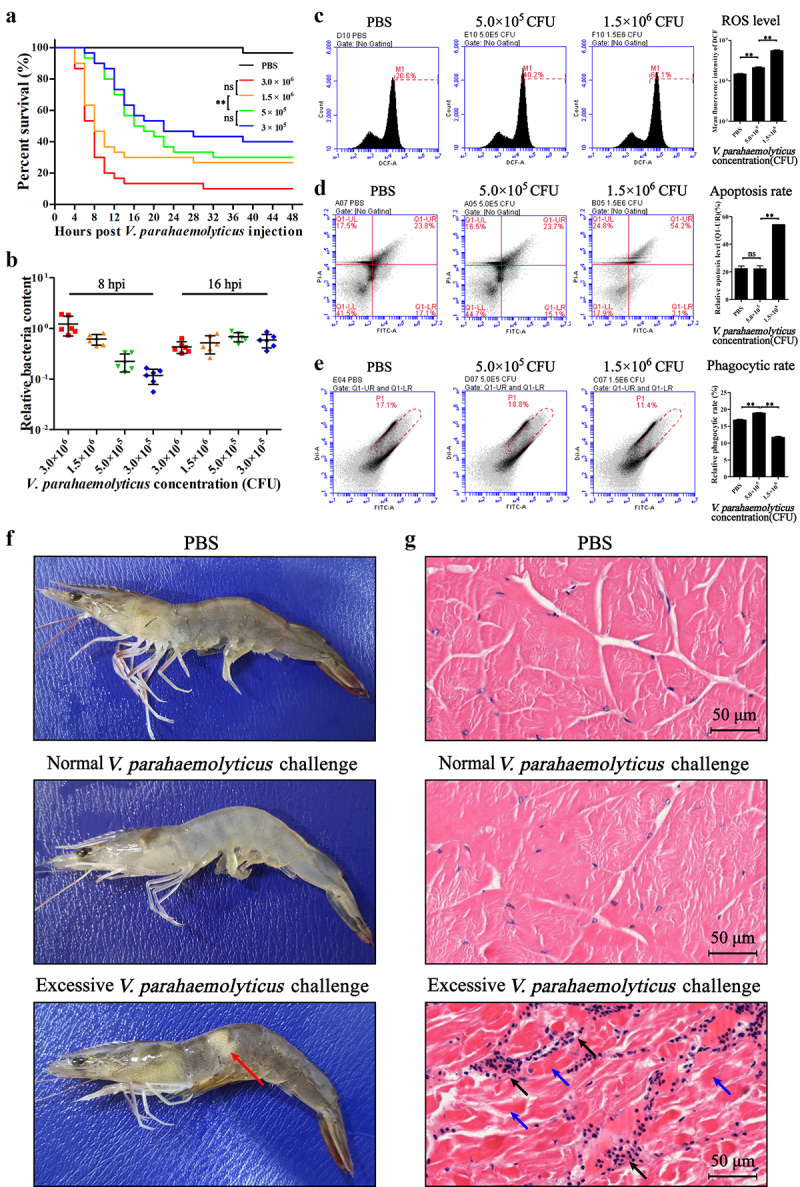
*V. parahaemolyticus* infection in *P. vannamei*. Shrimp were challenged with PBS (as control), 3.0 × 10^6^, 1.5 × 10^6^, 5.0 × 10^5^, and 3.0 × 10^5^ CFU *V. parahaemolyticus*. (a) the survival rates of shrimp after infection. (b) The levels of *V. parahaemolyticus* in tissues at 8 and 16 hpi analyzed by qPCR. (c, d) the pathological appearance and muscle tissue examination (H.E. staining) of shrimp infected with PBS (control), low-dose (5.0 × 10^5^ CFU) and high-dose (1.5 × 10^6^ CFU) *V. parahaemolyticus*. Red arrows, the opaque and whitish injection area of muscle tissue. Blue arrows, the muscle fibers arranged in bundles. Black arrows, the hemocytes infiltrating into the gaps between muscle bundles. The hemocytes from infected shrimp were subjected to flow cytometry analyses for intracellular ROS (e), apoptosis (f), and phagocytosis (g). *ns*, *p* > 0.05; **, *p* < 0.01.

The acute *Vibrio* infection under natural conditions generally occurs in water containing high bacterial concentration [33,34]. In this study, with the increase of *V. parahaemolyticus* doses, the survival rate of shrimp decreased significantly and the death time of shrimp was greatly advanced (Figure 1a). Shrimp challenged with high doses of bacteria (3.0 × 10^6^ and 1.5 × 10^6^ CFU) began to die at 4 h post injection (hpi) and their survival rates rapidly declined to lower levels than those of shrimp infected with low doses (5.0 × 10^5^ and 3.0 × 10^5^ CFU) (Figure 1a). The bacterial loads in tissues at 8 hpi in the experimental groups were just correlated with the original injection doses but did not show significant difference at 16 hpi (Figure 1b). Unlike that in the low-dose *V. parahaemolyticus* (5.0 × 10^5^ CFU) infected shrimp, in the high-dose (1.5 × 10^6^ CFU) infected moribund shrimp, the injection area in muscle appeared opaque and whitish, where the muscle fibers arranged in bundles and the gaps were enlarged and infiltrated with hemocytes (Figure 1c and d). These suggested that the excessive *V. parahaemolyticus* challenge (EVC) with high bacterial doses caused more acute infection in shrimp than normal *V. parahaemolyticus* challenge (NVC) with low doses. For the hemocytes of shrimp in the EVC group, the intracellular reactive oxygen species (ROS) and apoptosis rate were significantly higher than those in the NVC group (Figure 1e,f), while the phagocytic activity was lower than that in the NVC group (Figure 1g).

### Overactivation of shrimp JAK-STAT pathway in acute bacterial infection

**Figure 2.**
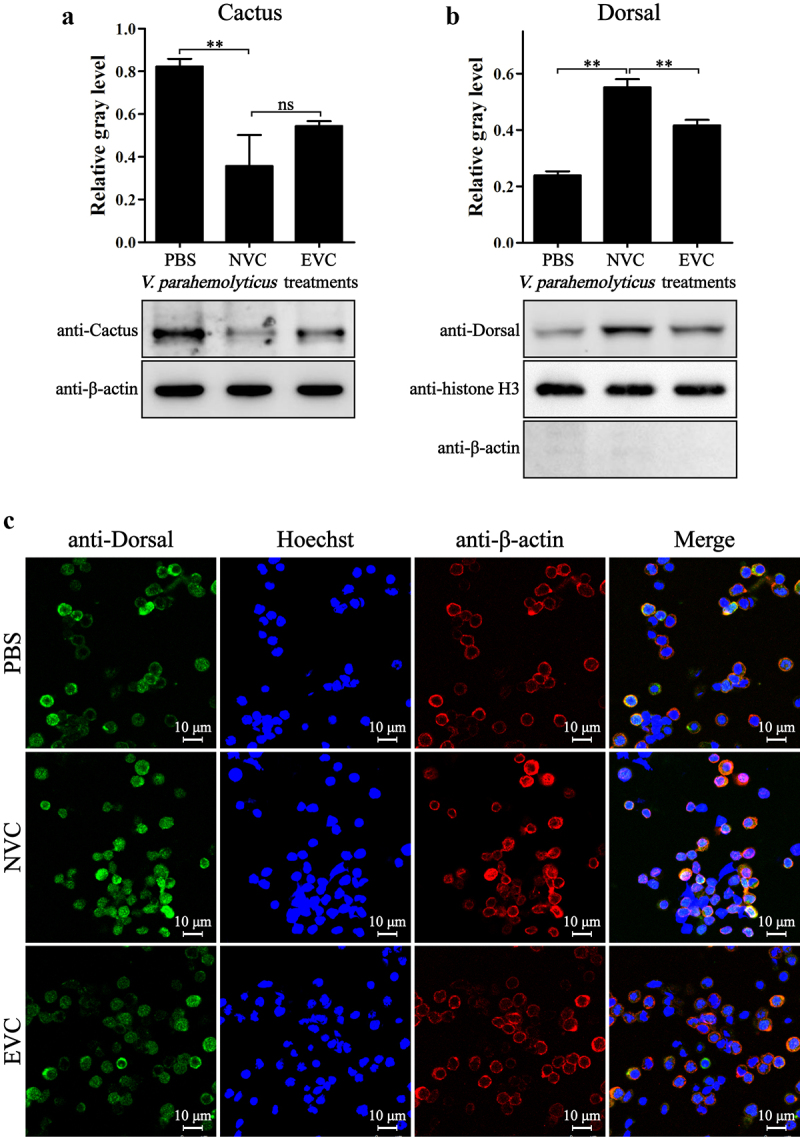
Activation of Dorsal in *V. parahaemolyticus-*infected shrimp. The protein levels of cytoplasmic Cactus (a) and nucleus-localized Dorsal (b) in hemocytes from EVC and NVC shrimp were analyzed by Western-blot. (c) Immunofluorescent analysis of the localization of Dorsal (green) in hemocytes from infected shrimp. The cytoplasm was marked with red fluorescence by detection of β-actin and the nucleus was stained with Hoechst 33342 (blue).

**Figure 3.**
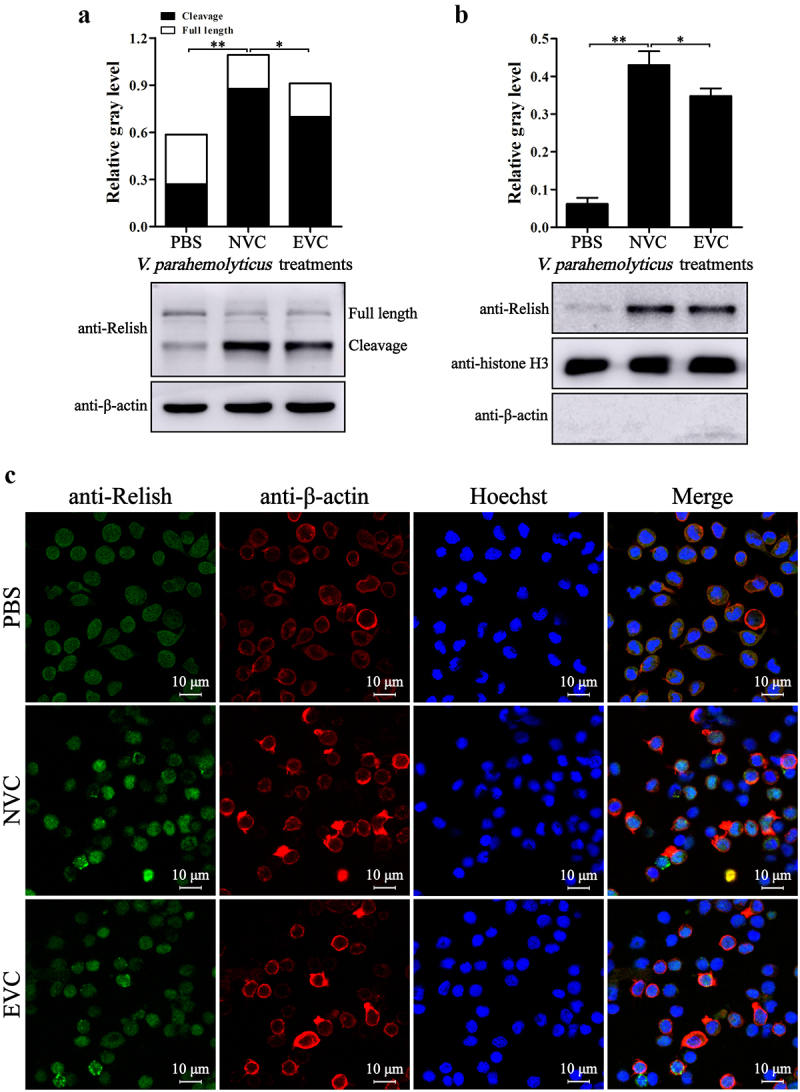
Activation of relish in *V. parahaemolyticus-*infected shrimp. Western-blot analyses of the full length and cleavage form (activated form) of Relish (a) and nucleus-localized Relish (b) in hemocytes from EVC and NVC shrimp. (c) Immunofluorescent analysis of the localization of Relish (green) in hemocytes from infected shrimp. The cytoplasm was marked with red fluorescence by detection of β-actin and the nucleus was stained with Hoechst 33342 (blue).

**Figure 4.**
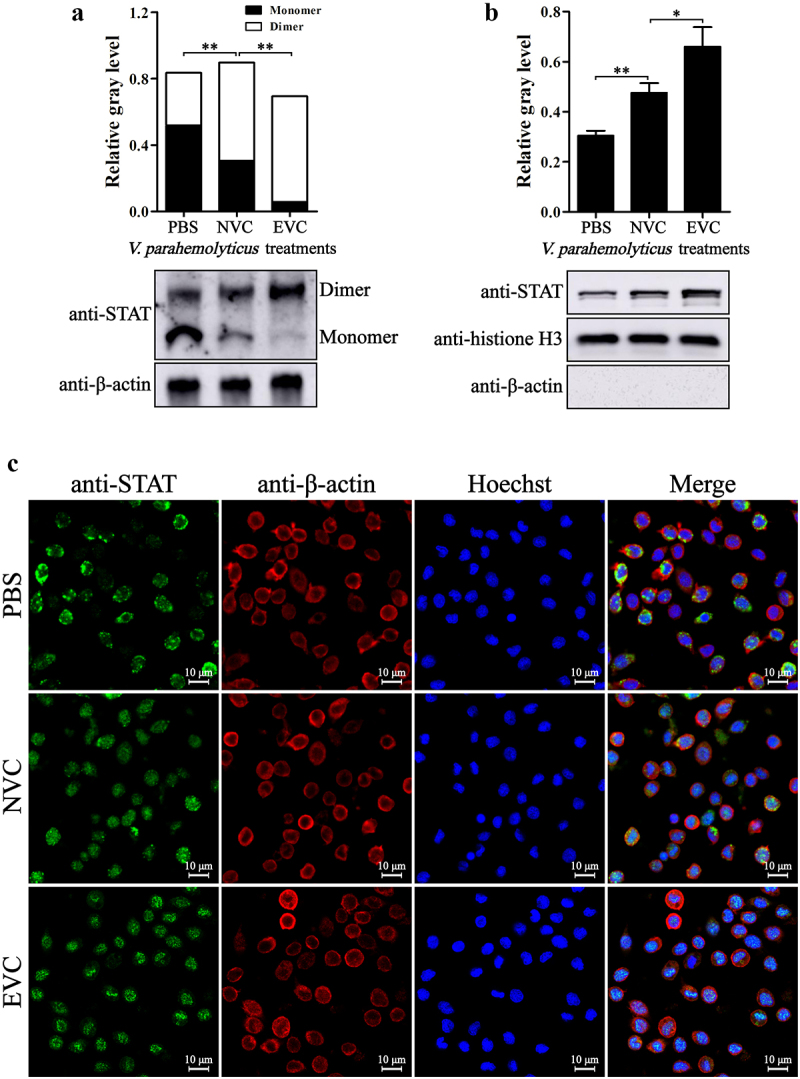
Activation of JAK-STAT pathway in *V. parahaemolyticus* infected shrimp. The dimerization (a) and nuclear translocation (b) of STAT in hemocytes from EVC and NVC shrimp were analyzed by Western-blot of native-PAGE and SDS-PAGE, respectively. (c) Immunofluorescent analysis of the localization of STAT (green) in hemocytes from infected shrimp. The cytoplasm was marked with red fluorescence by detection of β-actin and the nucleus was stained with Hoechst 33342 (blue).

The shrimp immunity is largely controlled by the JAK-STAT, Dorsal, and Relish pathways. As acute *V. parahaemolyticus* infection led to overactivation of immunity, the activation states of these pathways in EVC and NVC shrimp were then investigated. After *V. parahaemolyticus* infection, the hallmarks of Dorsal pathway activation, i.e. degradation of Cactus (Figure 2a) and nuclear translocation of Dorsal (Figure 2b,c), were significantly enhanced, but the activation degree of Dorsal pathway in EVC shrimp was reduced compared with that in NVC shrimp. Similarly, the activation of Relish pathway, characterized by cleavage and nuclear translocation of Relish, in EVC shrimp was also weaker than that in NVC shrimp (Figure 3a–c). In contrast, the levels of STAT dimerization and nuclear translocation in EVC shrimp were significantly higher than those in NVC shrimp (Figure 4a–c), indicating an excessive activation of the JAK-STAT signaling caused by acute *V. parahaemolyticus* infection.

## The role of JAK-STAT signaling in acute Vibrio disease

**Figure 5.**
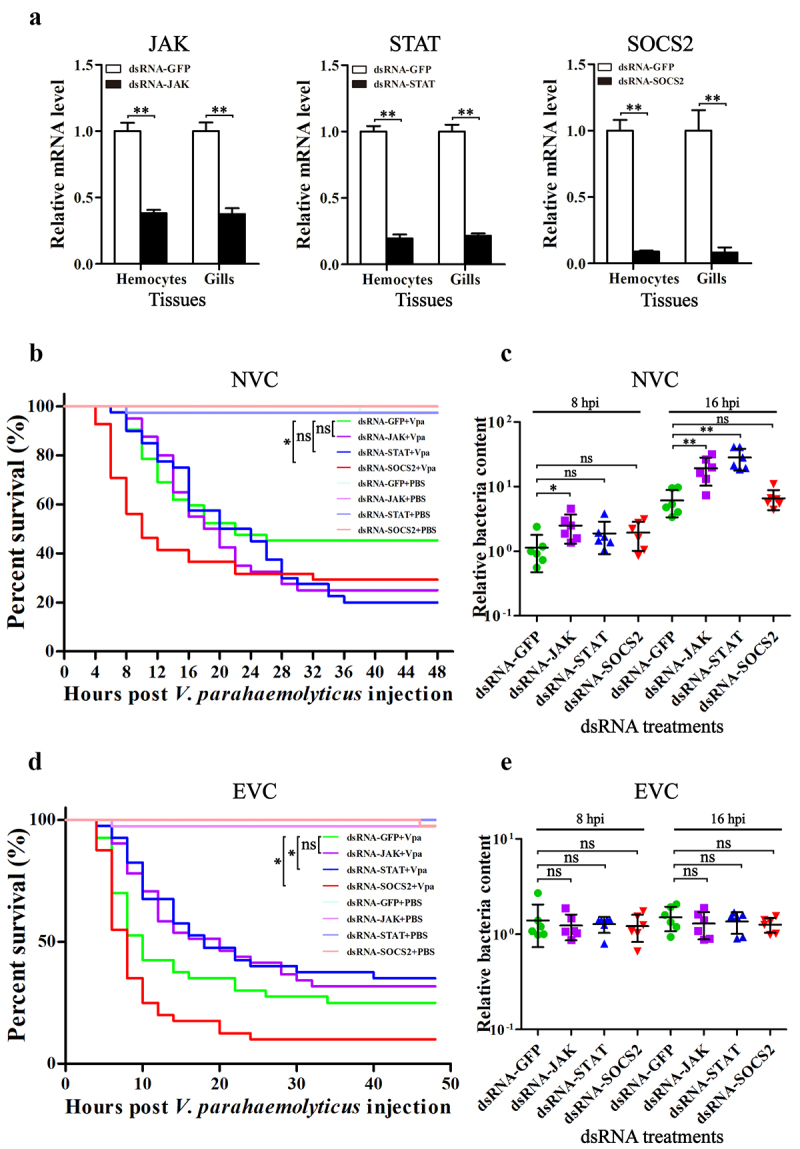
The roles of JAK-STAT pathway in *V. parahaemolyticus* infection. (a) qPCR evaluation of the RNAi efficiencies of *JAK*, *STAT* and *SOCS2 in vivo* (*n* = 9). (b, c) the survival rates of the dsRNA-treated shrimp after infection with 5.0 × 10^5^ and 1.5 × 10^6^ CFU of *V. parahaemolyticus*. (d, e) the bacterial loads in gills analyzed by qPCR. *ns*, *p* > 0.05; *, *p* < 0.05; **, *p* < 0.01.

To explore the role of the JAK-STAT pathway in acute infection, the *JAK*, *STAT,* and *SOCS2* genes were silenced using RNAi strategy. Injection of specific dsRNAs significantly suppressed the expression of these genes (Figure 5a). In the NVC group (infected with 5.0 × 10^5^ CFU *V. parahaemolyticus*), silencing of *JAK* and *STAT* increased the mortality of shrimp and elevated the bacterial load in tissues, although the differences between the mortality curves did not reach significance, but the silencing of *SOCS2* showed opposite results (Figure 5b, c). Most deaths of the *SOCS2*-dsRNA treated shrimp occurred within 4–8 hpi, earlier than those of the GFP-dsRNA treated control, indicating that enhancing the activation of JAK-STAT signaling could transform normal infection into acute infection in shrimp. In contrast, in the EVC group (infected with 1.5 × 10^6^ CFU *V. parahaemolyticus*), *STAT* silencing significantly decreased the mortality of shrimp. Similar results were observed after *JAK* silencing, although the mortality curves showed no significance. Silencing of *SOCS2* also promoted the death of shrimp, leading to a higher mortality than the control (Figure 5d). However, there was no difference in tissue bacterial loads between these EVC groups (Figure 5e), indicating that the difference in mortality may not be related to the replication of bacteria *in vivo*. These suggested that the JAK-STAT pathway plays different roles in normal and acute infections. Excessive activation of JAK-STAT essentially contributes to the pathogenesis of acute bacterial infection in shrimp.

**Figure 6.**
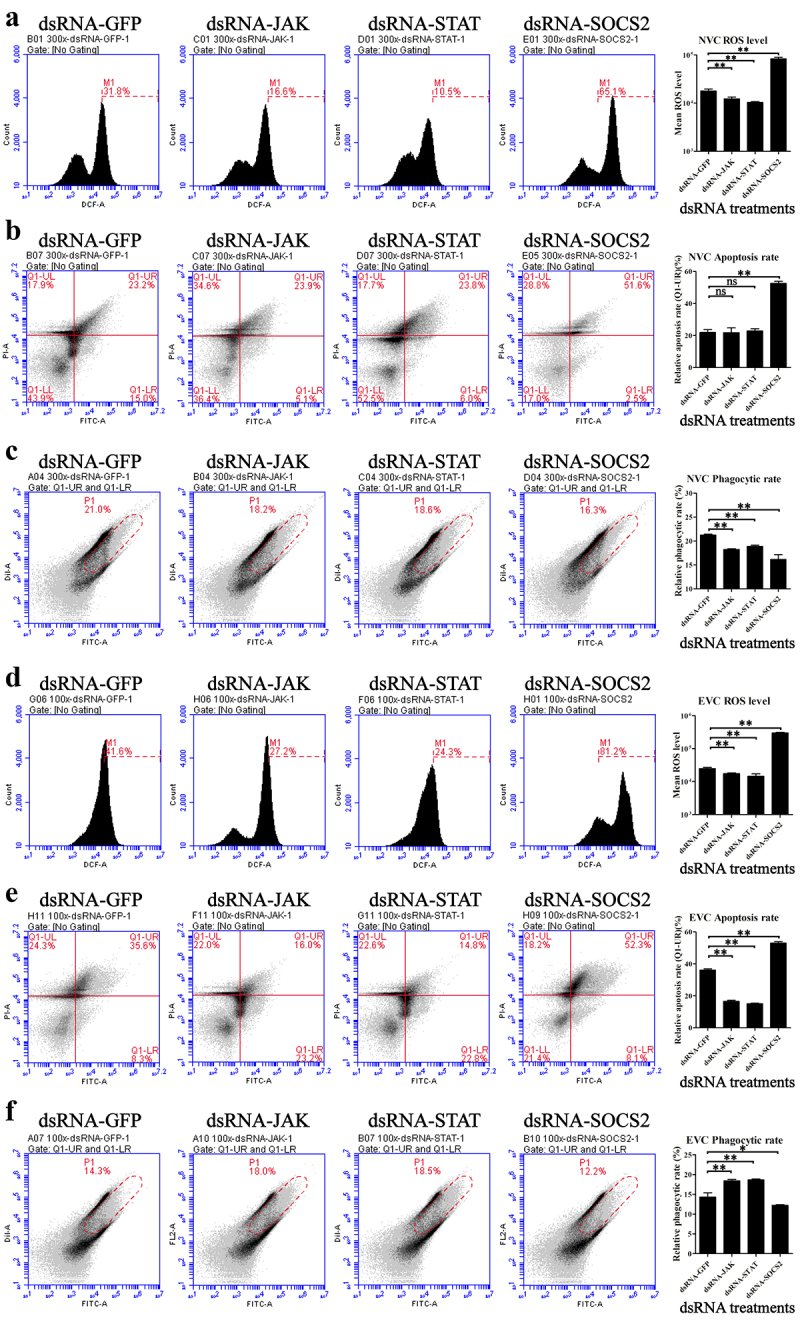
Influence of JAK-STAT pathway on hemocytes from *V. parahaemolyticus*-challenged shrimp. The intracellular ROS (a, d), apoptosis (b, e), and phagocytosis (c, f) of hemocytes in *JAK*-, *STAT* and *SOCS2*-silenced shrimp after infection with 5.0 × 10^5^ and 1.5 × 10^6^ CFU of *V. parahaemolyticus* were analyzed using flow cytometry. Hemocytes for each treatment were from nine shrimp. *ns*, *p* > 0.05; *, *p* < 0.05; **, *p* < 0.01.

In NVC shrimp, inhibition of *JAK* and *STAT* decreased the ROS level in hemocytes, while inhibition of *SOCS2* exhibited an opposite result (Figure 6a). The apoptosis rate of hemocytes from NVC shrimp did not change after *JAK* or *STAT* silencing but increased after *SOCS2* silencing (Figure 6b). Furthermore, *JAK*, *STAT,* and *SOCS2* have consistent activation effects on the phagocytosis of NVC shrimp hemocytes (Figure 6c). After *SOCS2* silencing, the phagocytic activity of hemocytes decreased more than that after *JAK* and *STAT* silencing. In contrast, for the EVC shrimp hemocytes, the ROS and apoptosis rate were decreased after *JAK* and *STAT* silencing and increased after *SOCS2* silencing (Figure 6d, e), whiles the phagocytic activity showed an opposite trend (Figure 6f).

## Discussion

The current study showed that high-doses of *V. parahaemolyticus* led to acute infection in shrimp with a significantly earlier death time compared to low-doses. The bacterial load of *V. parahaemolyticus* in tissues during acute infection varied slightly compared to low-dose infection, suggesting that the rapid death of acutely infected shrimp may not be solely due to the virulence of *V. parahaemolyticus*. In shrimp suffering acute infection, a large number of hemocytes in the opaque and whitish muscle tissue were observed, similar to the sign of macrophage and neutrophil infiltration in mammalian inflammatory tissues. The changes of intracellular ROS, apoptosis rate, and phagocytic activity of hemocytes in acutely infected shrimp also resemble the signs of innate immune cell activation during inflammation in mammals [35–37]. On the other hand, attenuation of immune overactivation alleviated the severity of acute *V. parahaemolyticus* infection and decreased the mortality of shrimp, indicating the involvement of overactivated immune response in the pathogenesis of acute bacterial infection in crustaceans. More importantly, this study suggests that infected invertebrates also show a pathological sign similar to mammalian inflammation, which is important for the onset and progression of infectious diseases, especially in the context of acute infection.

In general, immune regulation is balanced between activating and inhibitory signaling pathways that define the defense against invading pathogens to maintain immune homeostasis [38]. Aberrant activation of immune signaling is the intrinsic cause of uncontrolled immune responses, especially in the context of acute infection, which has been extensively studied in mammals. The JAK-STAT pathway is critical for establishing the inflammatory response by activating expression of pro-inflammatory cytokines, such as IL-1β, IL-6, and MCP-1, in mammals [39–41]. In human, targeting the components of JAK-STAT pathway has been a potential strategy for the treatment of inflammatory diseases [10]. In *Drosophila*, the JAK-STAT pathway is also known to be essentially involved in the mechanical stress-induced acute inflammation [42]. In this study, overactivation of JAK-STAT pathway was observed in *Vibrio* acutely infected shrimp, characterized by high levels of STAT phosphorylation and nuclear translocation compared with low-dose infection. Suppression of JAK-STAT signaling by RNAi strategy reduced the clinical signs and mortality of acutely infected shrimp. On the other hand, enhancing the JAK-STAT signaling activation by silencing the inhibitor *SOCS2* transformed normal infection into acute infection. This suggests that overactivation of the JAK-STAT pathway is an important factor contributing to the pathological changes and mortality in acute bacterial-infected shrimp. Moreover, the bacterial endotoxin genes *pirA*^*vp*^ and *pirB*^*vp*^ have been shown to be associated with the lethal diseases caused by *Vibrio* infection in shrimp [18,19,43]. However, the mechanism of lethality of these endotoxins is not yet fully understood. A recent study has shown that recombinant *Vibrio parahaemolyticus* PirA and PirB proteins have an important impact on the immune response of shrimp [44]. Whether *Vibrio* endotoxins PirA and PirB achieve pathological and lethal effects in shrimp by affecting the inflammatory response and the activation of the JAK-STAT pathway deserves further investigation.

The NF-κB family members are conserved across invertebrates and mammals and serve as downstream transcription factors of multiple cellular immune signaling cascades that are critical for immune regulation. Shrimp have two NF-κB family members, Dorsal and Relish. Dorsal is structurally similar to the Rel subfamily of mammal NF-κBs and can be activated upon the degradation of its cytoplasm inhibitor Cactus [45,46]. In contrast, Relish is similar to NF-κB1 (p105) and NF-κB2 (p100) of mammals, which is activated by protein cleavage to release the N-terminus region for nuclear translocation [47]. The Relish and Dorsal signaling pathways are essentially involved in immune responses against bacterial and viral infections in shrimp. It has been reported that in mammals, NF-κB family members play key roles in orchestrating the multiple mechanistic aspects of inflammatory response by regulating the expression of a large number of inflammation-related genes [48]. Overactivation of NF-κB causes immoderate expression of pro-inflammatory factors, such as nitric oxide, TNF-α, IL-1β, and IL-6, resulting in tissue damage [49,50]. However, the current study showed that compared to normal infection, the activation levels of Dorsal and Relish were even decreased in inflammation due to acute *V. parahaemolyticus* infection in shrimp. That is, acute infection by *Vibrio* did not cause overactivation of NF-kB, but rather inhibited it to some extent. The roles of NF-kB signaling in acute *Vibrio* diseases require further investigation. A previous study indicates that the JAK-STAT pathway could suppress the activation of Dorsal by regulating the expression of miRNA-1, which targets the cellular adaptor MyD88 upstream of the Dorsal pathway [51]. Therefore, the suppression of Dorsal activity in the context of acute *Vibrio* infection may result indirectly from overactivation of JAK-STAT signaling. This suggests that the acute inflammation in shrimp is associated with the interaction and balance of multiple signaling pathways, warranting further in-depth investigation.

Taking together, the current study suggests that the pathological sign of inflammation also occurs in shrimp after bacterial infection. Like that in mammals, the inflammation triggered by immune overreaction is a major cause of death from infection in invertebrates. The JAK-STAT pathway plays a positive role in antibacterial response in low-dose *V. parahaemolyticus* infection but is overactivated in acute infection leading to accelerated death of shrimp. Responses to acute infection that exceed the regulatory extent of this mechanism could lead to overactivation of the immune system, resulting in pathological changes in shrimp that are similar to those of acute inflammatory responses in mammals. Therefore, this study suggests that the pathogenicity of *Vibrio* to crustaceans is not only related to its own virulence but also to its over-activating effect on the host immune system, which should be taken into account in the prevention and treatment of aquatic vibriosis.

## Supplementary Material

Figure S1.jpg

Supplementary Table S1.docx

## Data Availability

The data supporting the findings of this study are available on https://doi.org/10.6084/m9.figshare.28014407
