# Supplementary figures and images for: Excessive activation of JAK-STAT signaling contributes to inflammation induced by acute Vibrio infection in shrimp
Source: Virulence. 2025 Jan 17;16(1):2451169. doi: 10.1080/21505594.2025.2451169 (PMC11749392; doi:10.1080/21505594.2025.2451169)

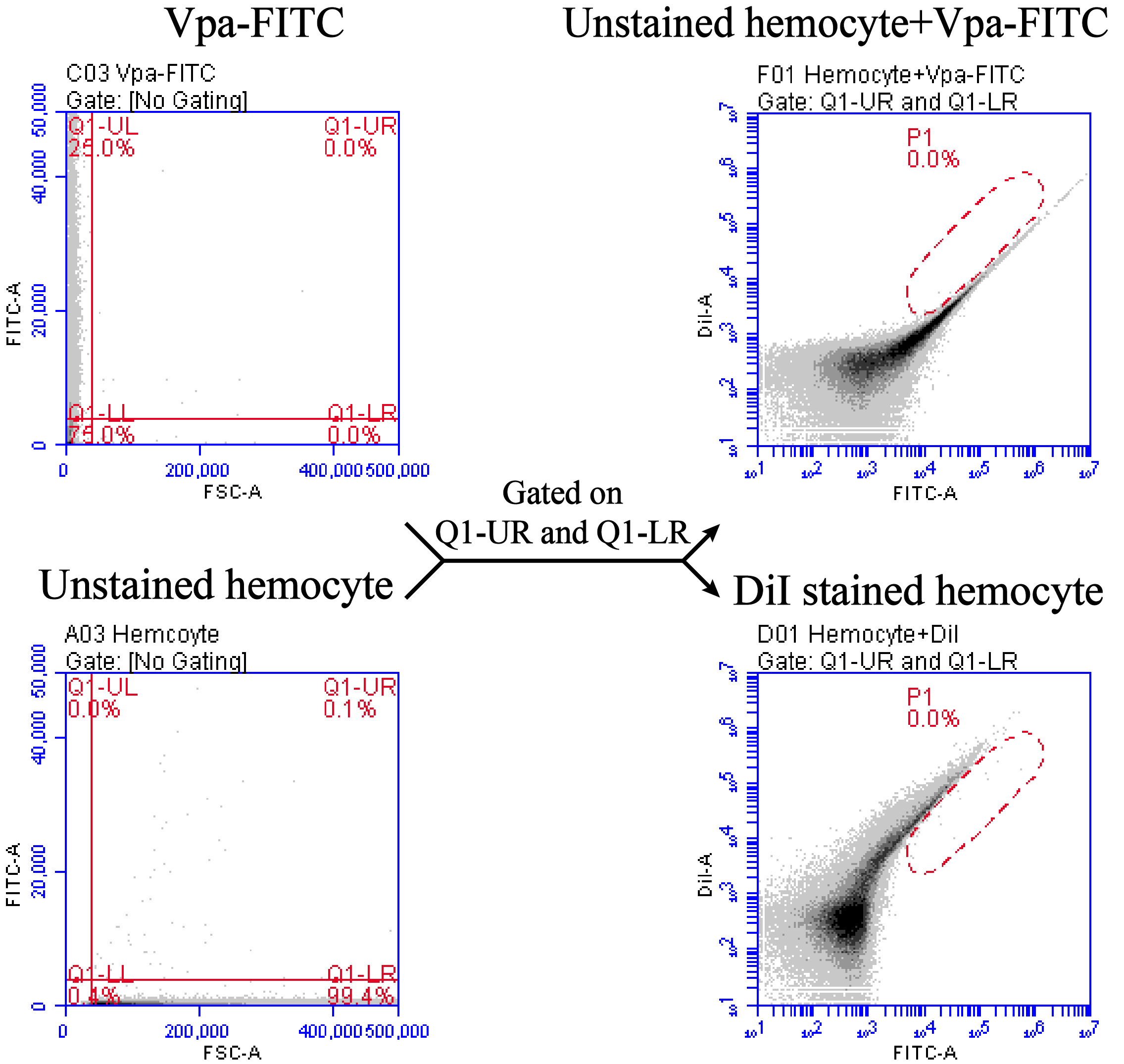

Supplement: Figure S1.jpg [file KVIR_A_2451169_SM9643.jpg]
