# Supplementary material for: Excessive activation of JAK-STAT signaling contributes to inflammation induced by acute Vibrio infection in shrimp
Source: Virulence. 2025 Jan 17;16(1):2451169. doi: 10.1080/21505594.2025.2451169 (PMC11749392; doi:10.1080/21505594.2025.2451169)
Supplement: Supplementary Table S1.docx [file KVIR_A_2451169_SM9642.docx]

**Supplementary Table S1 Primer sequences**

**Primers Sequences (5’ to 3’)**

| **dsRNA synthesis** | |
| --- | --- |
| JAK-dsT7F | GGATCCTAATACGACTCACTATAGGGCCTACAACAATGTCTACGCTG |
| JAK-dsT7R | GGATCCTAATACGACTCACTATAGGACATTGCGAGCTGCAAGATC |
| JAK-dsF | GCCTACAACAATGTCTACGCTG |
| JAK-dsR | ACATTGCGAGCTGCAAGATC |
| STAT-dsT7F | GGATCCTAATACGACTCACTATAGGATTACTCGTCTCTTGTCATCACTG |
| STAT-dsT7R | GGATCCTAATACGACTCACTATAGGGCATTATCCCATGACACTGTCG |
| STAT-dsF | ATTACTCGTCTCTTGTCATCACTG |
| STAT-dsR | GCATTATCCCATGACACTGTCG |
| SOCS2-dsT7F | GGATCCTAATACGACTCACTATAGG CACTTCTGGCGGAGGCTTAG |
| SOCS2-dsT7R | GGATCCTAATACGACTCACTATAGG CATGTGCTTCTGGCTGTGAC |
| SOCS2-dsF | CACTTCTGGCGGAGGCTTAG |
| SOCS2-dsR | CATGTGCTTCTGGCTGTGAC |
| GFP-dsT7F | GGATCCTAATACGACTCACTATAGGATGGTGAGCAAGGGCGAGGAG |
| GFP-dsT7R | GGATCCTAATACGACTCACTATAGGTTACTTGTACAGCTCGTCCATGCC |
| GFP-dsF | ATGGTGAGCAAGGGCGAGGAG |
| GFP-dsR | TTACTTGTACAGCTCGTCCATGCC |
| **qRT-PCR** | |
| pirA^VP^-qRTF | ACTATTCTCACGATTGGACTGTC |
| pirA^VP^-qRTF | TCTACACTACGACCGACTTCC |
| STAT-qRTF | GAATGGTGTGAAGCACTAGCAG |
| STAT-qRTR | GTCGGATAGAGTCAATGTCGTG |
| JAK-qRTF | GCGCAAGTTCATCAACAACAG |
| JAK-qRTR | CGCAAGTTCATCAACAACAGAA |
| SOCS2-qRTF | CCAAACGCCCACTTCATCC |
| SOCS2-qRTR | CTGAGCCTGCCACACAATG |
| EF-1α-qRTF | TATGCTCCTTTTGGACGTTTTGC |
| EF-1α-qRTR | CCTTTTCTGCGGCCTTGGTAG |
